# Auditory Processing Disorder in childhood: a critical appraisal of diagnostic validity, functional assessment, and interdisciplinary practice

**DOI:** 10.3389/fnhum.2026.1715787

**Published:** 2026-04-08

**Authors:** Júlio César Claudino dos Santos, Marco Antonio Arruda, Marcelo Rodrigues Masruha

**Affiliations:** 1 Laboratório de Neurologia Experimental, Programa de Pós-graduação em Ciências da Saúde, Universidade do Extremo Sul Catarinense, UNESC, Criciúma, Brazil; 2 Universidade Federal Fluminense (UFF), Niterói, Rio de Janeiro, Brazil; 3 Programa de Pós-Graduação em Neurologia e Neurociência, Universidade Federal Fluminense, UFF, Niterói, Brazil; 4 Instituto Glia – Neurologia e Neurodesenvolvimento, Ribeirão Preto, Brazil; 5 Instituto de Neurociência do Espírito Santo, Vitória, Brazil

**Keywords:** auditory perceptual disorders, child, diagnosis, evidence-based practice, neurodevelopmental disorders

## Abstract

Auditory Processing Disorder (APD) refers to listening difficulties in individuals with normal hearing thresholds but impaired processing of auditory information. Despite decades of research, its diagnostic validity in childhood remains highly debated. This narrative review critically examines the conceptual foundations, methodological limitations, and clinical implications of APD, with the goal of promoting more functional and interdisciplinary approaches to auditory assessment. National and international literature, including professional guidelines, systematic reviews, and observational studies, was reviewed to analyze diagnostic definitions, criteria, assessment instruments, overlap with other neurodevelopmental disorders, and implications for clinical decision-making. The findings reveal considerable heterogeneity across diagnostic criteria and testing protocols, leading to wide variability in reported prevalence. Many assessment tools were originally designed for adults and lack developmental calibration or psychometric validation for children, which limits diagnostic reliability. In addition, there is significant overlap between APD and other neurodevelopmental conditions such as developmental language disorder, dyslexia, attention-deficit/hyperactivity disorder, and learning difficulties, which challenges the specificity and clinical validity of the construct. Although international guidelines recognize these inconsistencies, their implementation in clinical practice remains fragmented, producing variable and sometimes inconclusive diagnostic outcomes. Current evidence provides limited support for APD as a distinct diagnostic entity in childhood. A shift toward functional, ecologically valid, and interdisciplinary assessment is needed. Integrating behavioral, cognitive, linguistic, and neurophysiological measures may improve the ability to differentiate developmental variability from genuine auditory dysfunction. Such an approach emphasizes functionality over categorization, supports ethical and evidence-based clinical practice, and enhances the precision and effectiveness of interventions for children with listening difficulties.

## 1 Introduction

Hearing assessment in children traditionally relies on pure tone audiometry combined with complementary measures such as tympanometry, stapedial reflexes, and otoacoustic emissions (Forli et al., 2025; Hall, 2016). While these procedures are fundamental for identifying peripheral hearing loss, they do not necessarily capture how children process complex sounds in real-life settings such as classrooms or noisy environments (Iliadou and Kiese-Himmel, 2018). To address this limitation, the concept of Central Auditory Processing Disorder (CAPD) emerged, describing difficulties in perceiving and interpreting auditory information despite normal hearing thresholds. These difficulties are thought to interfere with communication, language development, academic performance, and social interaction [British Society of Audiology (BSA), 2018].

Over the past decades, scientific and clinical interest in auditory processing disorders has increased substantially, leading to the publication of multiple guidelines and position statements by professional bodies such as the American Speech-Language-Hearing Association (ASHA) (2005), American Academy of Audiology (2010), and various European consortia (Samara et al., 2023). Despite these initiatives, there remains no consensus regarding the construct’s validity, diagnostic criteria, or clinical applicability (DeBonis, 2017a; Neijenhuis et al., 2019). Reported prevalence rates vary widely, with studies showing diagnostic outcomes ranging from 7 to 93% in similar populations depending on the criteria and tests applied (Wilson and Arnott, 2013). Such discrepancies highlight the fragility of the construct and the lack of reproducibility across laboratories and countries.

In recent years, international terminology has evolved. The expression Central Auditory Processing Disorder (CAPD), which initially emphasized presumed central nervous system dysfunction, has gradually been replaced by Auditory Processing Disorder (APD), reflecting a broader and less localization-based framework (De Wit et al., 2018; Neijenhuis et al., 2019). More recently, the term listening difficulties (LiD) has been introduced to encompass a wider range of auditory and cognitive mechanisms that contribute to real-world listening performance (Moore, 2018, Moore et al., 2025). This conceptual evolution recognizes that auditory perception in complex environments depends on the integration of bottom-up sensory and top-down cognitive processes. In the present review, we adopt APD as the primary terminology, while acknowledging the historical equivalence of CAPD and the relevance of the broader construct of LiD.

A major source of uncertainty arises from the instruments used to diagnose APD. Many of the behavioral tests currently employed were originally developed for adults with acquired brain injury and lack psychometric validation for pediatric populations (Moore et al., 2018). These tests impose significant linguistic, attentional, and memory demands, making it difficult to distinguish auditory deficits from other neurodevelopmental conditions such as developmental language disorder (DLD), dyslexia, or ADHD (De Wit et al., 2018; Tomlin et al., 2015). Systematic reviews have consistently demonstrated overlapping features between children labeled with APD and those with other developmental disorders, challenging the specificity and clinical relevance of the diagnosis (De Wit et al., 2016; Neijenhuis et al., 2019).

This lack of conceptual and methodological clarity carries important clinical and ethical implications. Fragile or inconsistent diagnostic criteria may delay appropriate interventions, promote unnecessary medicalization, and divert educational or therapeutic resources (Vermiglio, 2014; Spaulding et al., 2006). Consequently, there is a growing need to re-evaluate the diagnostic validity of APD in childhood and to reconsider how auditory difficulties should be conceptualized and managed within an evidence-based, interdisciplinary framework. The present narrative review critically examines the theoretical, methodological, and clinical foundations of APD, aiming to identify the main sources of diagnostic inconsistency and to discuss alternative approaches for functional and ecologically valid assessment.

## 2 Method

Although this work follows a narrative review format, methodological transparency was ensured to enhance rigor, reproducibility, and clarity of scope. The process was guided by the SANRA (Scale for the Assessment of Narrative Review Articles) framework (Baethge et al., 2019).

The review focused on studies addressing the conceptual validity, diagnostic variability, and psychometric properties of Auditory Processing Disorder (APD) in childhood. Databases were searched using predefined terms, and evidence was thematically synthesized into four analytical domains: diagnostic definitions, test validity, overlap with other neurodevelopmental disorders, and guideline quality (see Table 1).

**Table 1:** Overview of the narrative review methodology.

Component	Description
Type of review	Narrative review—structured according to SANRA guidelines (Baethge et al., 2019)
Databases searched	PubMed/MEDLINE, Scopus, Web of Science
Time frame	January 2010—January 2025
Search strategy	(“auditory processing disorder” OR “central auditory processing disorder” OR “listening difficulties”) AND (“child” OR “pediatric”) AND (“diagnosis” OR “assessment” OR “validity” OR “guideline” OR “neurodevelopment”)
Supplementary sources	Reference lists of key articles and professional guidelines (ASHA, AAA, BSA, ESLA)
Inclusion criteria	(a) Peer-reviewed studies on diagnostic validity or test reliability in children; (b) systematic, scoping, or narrative reviews; (c) guidelines or consensus statements; (d) observational studies on overlap with language, reading, or attention disorders
Exclusion criteria	(a) Adult or acquired brain injury studies; (b) intervention-only trials; (c) non-peer-reviewed or gray literature
Thematic domains for synthesis	1. Definitions and diagnostic criteria; 2. Psychometric properties of tests; 3. Overlap with other neurodevelopmental disorders; 4. Guideline quality and clinical implications
Data synthesis approach	Qualitative and thematic synthesis emphasizing convergence/divergence among sources, cross-cultural perspectives, and ecological validity
Quality and transparency criteria	Guided by SANRA standards: justification of topic relevance, comprehensive search, logical structure, critical interpretation, appropriate referencing, and clear presentation

## 3 Lack of unified diagnostic criteria

Despite decades of international research, there remains no consensus on how Auditory Processing Disorder (APD) in children should be defined or diagnosed. Variability in terminology, inclusion criteria, and diagnostic thresholds across professional organizations has generated considerable confusion. Different bodies—including the American Speech-Language-Hearing Association (ASHA) (2005), American Academy of Audiology (2010), and British Society of Audiology (2018)—have published guidelines that differ markedly in conceptual focus, number and type of behavioral tests recommended, and criteria for diagnostic confirmation. More recently, the ESLA (European Society for Language and Communication Disorders) (2021) has proposed updates emphasizing the integration of cognitive and linguistic factors.

While these documents have advanced the field, their heterogeneity results in inconsistent diagnosis rates, with prevalence estimates ranging from 2 to 7% in population studies to over 90% in specialized clinical samples (Wilson and Arnott, 2013). The absence of standardized criteria has contributed to diagnostic inflation in some contexts and underidentification in others. According to Neijenhuis et al. (2019), even when similar tests are used, small variations in cut-off values or interpretation rules can drastically alter diagnostic outcomes, underscoring the fragility of current operational definitions.

Evaluation of guideline quality using international appraisal frameworks such as the AGREE II Instrument (Brouwers et al., 2010) and the Institute of Medicine (IOM) Standards for Developing Trustworthy Clinical Practice Guidelines (2011) reveals several weaknesses. Most APD guidelines lack transparent methodology, do not grade the strength of evidence, and rely heavily on expert consensus rather than systematic literature review. Stakeholder participation, conflict-of-interest management, and explicit implementation strategies are also rarely reported. These gaps limit reproducibility and hinder international harmonization of APD assessment protocols.

Beyond methodological inconsistency, terminological shifts add further complexity. The historical term Central Auditory Processing Disorder (CAPD) emphasized presumed central nervous system involvement, whereas the contemporary term Auditory Processing Disorder (APD) encompasses a broader, functionally oriented perspective. Recent literature, however, increasingly employs the concept of listening difficulties (LiD), acknowledging that many children experience listening problems in daily life without meeting strict diagnostic thresholds (Moore, 2018, Moore et al., 2025; Samara et al., 2023). This evolution reflects a growing recognition that APD often coexists with linguistic, attentional, and cognitive challenges, blurring the line between a discrete auditory disorder and broader neurodevelopmental variability.

To achieve diagnostic reliability and clinical utility, future frameworks should adopt transparent, evidence-based criteria, establish cross-cultural normative data, and evaluate the ecological validity of assessment tools. International collaboration following AGREE II and IOM standards could facilitate the development of unified guidelines that better align diagnostic practices with empirical evidence and real-world listening outcomes (see Table 2).

**Table 2:** International guidelines and key diagnostic differences in childhood Auditory Processing Disorder (APD).

Organization/guideline	Year	Diagnostic focus	Core tests recommended	Diagnostic threshold	Evidence base/quality (appraisal)
ASHA technical report	2005	Functional auditory processing deficits with normal audiogram	≥2 behavioral tests below 2 SD	≥2 SD below the mean on two tests	Expert consensus; limited psychometric validation
AAA clinical practice guidelines	2010	Central auditory pathway disorders	Behavioral battery + physiologic measures (ABR, MLR)	Performance ≥2 SD below mean on ≥2 tests	Expert panel; evidence graded informally
BSA position statement	2018	Listening difficulties with functional impact	Behavioral + questionnaire-based assessment	Not standardized (≥2 SD recommended)	Expert consensus; limited validation data
ESLA consensus report	2021	Integration of auditory and linguistic factors	Auditory tests + language and cognitive screening	Functional criteria linked to listening outcomes	Emerging framework; partial alignment with AGREE II
Summary	—	Conceptual and methodological heterogeneity across guidelines	Different test batteries and cut-offs lead to diagnostic variability	—	Most guidelines lack systematic evidence grading and stakeholder involvement

## 4 Psychometric and developmental limitations of assessment tools

A major limitation in the clinical assessment of Auditory Processing Disorder (APD) in children arises from the psychometric fragility and developmental inappropriateness of many diagnostic instruments currently in use. Most behavioral tests were originally designed for adults with acquired central lesions and were later adapted, often without sufficient validation, for pediatric populations (Moore et al., 2018; De Wit et al., 2018). Consequently, they impose linguistic, cognitive, and attentional demands that exceed the developmental level of many school-aged children, raising questions about whether poor performance reflects genuine auditory dysfunction or broader neurocognitive immaturity.

Test–retest reliability and construct validity remain critical weaknesses in the APD field. Many widely used measures, such as dichotic listening, temporal patterning, and speech-in-noise tests, exhibit moderate to low reliability coefficients in children (Neijenhuis et al., 2019; Dawes and Bishop, 2010; Drosos et al., 2024). Age norms are often derived from small or unrepresentative samples, limiting generalizability across linguistic and cultural contexts. Moreover, the lack of standardized scoring criteria leads to inconsistent interpretation of “abnormal” results, as diagnostic cut-offs vary across laboratories from one to two standard deviations below the mean. These issues compromise both the sensitivity and specificity of current test batteries and undermine their clinical reproducibility (Ahmmed et al., 2014; Alvand et al., 2022; Sharma et al., 2006).

Another fundamental problem is the ecological validity of APD tests—the extent to which laboratory measures predict real-world listening or learning performance. Studies increasingly show weak correlations between behavioral test outcomes and functional listening behaviors observed in classrooms or caregiver questionnaires (Sharma et al., 2022; Moore et al., 2025). This discrepancy suggests that many APD tasks may assess artificial constructs rather than meaningful auditory-cognitive functions relevant to daily life. The overreliance on laboratory-based stimuli may therefore contribute to overdiagnosis, especially among children with co-occurring language, attention, or memory difficulties (Samara et al., 2023).

From a developmental perspective, auditory processing abilities evolve in parallel with attention, working memory, and linguistic skills throughout childhood (Dawes et al., 2009). When tests designed for adults are administered to younger children, results may reflect developmental stage rather than disorder. This developmental overlap challenges the assumption that APD represents a discrete and static impairment. Longitudinal evidence indicates that auditory and cognitive measures interact dynamically over time, with many children who initially fail APD tests later achieving normal performance as cognitive-linguistic systems mature (Moore, 2018; Tomlin et al., 2015).

Given these psychometric and developmental challenges, there is growing advocacy for multimodal and interdisciplinary assessment frameworks. Integrating behavioral, electrophysiological, and functional listening measures may provide a more comprehensive and ecologically valid profile of children’s auditory abilities (Sharma and Purdy, 2020; Samara et al., 2023). Such approaches, combined with standardized international norms and validated caregiver/teacher questionnaires, could enhance diagnostic precision and reduce misclassification (see Table 3).

**Table 3:** Commonly used auditory processing tests and major psychometric/developmental limitations in pediatric populations.

Test category	Representative examples	Primary limitation	Developmental/validity concerns
Dichotic listening tests	Dichotic digits, competing sentences	Moderate reliability; high linguistic and attention load	Difficult to isolate auditory from cognitive deficits; poor cross-linguistic transferability
Temporal patterning/ordering tests	Frequency pattern, duration pattern	Limited normative data for young children	Require intact labeling and sequencing skills; confounded by speech output demands
Speech-in-noise/speech-in-competition tests	QuickSIN, HINT-C	Low ecological validity; influenced by language proficiency	Performance strongly affected by vocabulary, syntax, and working memory
Gap detection/temporal resolution tests	Random gap detection, GIN	Weak correlation with real-world listening	Task simplicity masks higher-order processing needs
Electrophysiological measures	ABR, MLR, P300	Provide neural indices but limited diagnostic specificity	Often normal in children labeled as APD; poor predictive value for functional outcomes

ABR, auditory brainstem response; MLR, middle latency response; GIN, gaps-in-noise test; HINT-C, hearing in noise test for children.

## 5 Neurocognitive overlap with other developmental disorders

One of the most debated issues in Auditory Processing Disorder (APD) research concerns its substantial overlap with other neurodevelopmental conditions, including developmental language disorder (DLD), dyslexia, and attention-deficit/hyperactivity disorder (ADHD). Empirical evidence indicates that many children diagnosed with APD also meet criteria for one or more of these conditions, challenging the specificity of APD as a discrete diagnostic entity (Dawes and Bishop, 2009; 2010; Konopka et al., 2024; Omidvar et al., 2023; Sanches et al., 2024; de Wit et al., 2016; Sharma and Purdy, 2020). This overlap extends across behavioral, neurocognitive, and neurophysiological levels, suggesting shared mechanisms rather than independent disorders.

From a behavioral standpoint, tasks commonly used to diagnose APD—such as dichotic listening, temporal sequencing, and speech-in-noise perception—rely heavily on linguistic comprehension, working memory, and attention control (Heine and O’Halloran, 2015). Children with DLD or dyslexia often perform poorly on these same tests, not necessarily because of auditory deficits but due to higher-order cognitive or linguistic demands (Dawes et al., 2009). Similarly, attention-related factors play a central role: in ADHD, inconsistent attention during auditory tasks can mimic APD-like performance patterns, especially on lengthy or repetitive test protocols (Gyldenkærne et al., 2014; Moore et al., 2025). Such findings raise concerns that many children labeled as APD may instead represent a heterogeneous mix of auditory, linguistic, and attentional vulnerabilities.

Neuroimaging research further supports the notion of overlapping neural substrates among these disorders. Functional MRI and electrophysiological studies suggest that listening difficulties frequently co-occur with alterations in neural systems supporting language, attention, and auditory processing, although the specificity of these findings to APD remains uncertain (Dawes and Bishop, 2009; Sharma and Purdy, 2020). Moreover, atypical top-down modulation of auditory cortex activity by prefrontal and parietal regions has been documented in both APD and ADHD (Moore et al., 2025), consistent with a shared deficit in auditory attention and executive control. These converging findings suggest that listening difficulties in many children may arise from distributed network dysfunctions rather than isolated impairments within the auditory system.

Emerging models increasingly conceptualize listening difficulties as multidimensional phenomena involving auditory, linguistic, attentional, and cognitive factors rather than as isolated auditory deficits (Moore, 2018; Dawes and Bishop, 2009). The “multifactorial model” proposes that auditory perception reflects dynamic interactions between bottom-up sensory encoding and top-down linguistic and cognitive regulation (Sharma et al., 2022). Within this framework, listening performance results from the integration of auditory fidelity, language proficiency, attention control, and working memory capacity. This integrative perspective provides a more ecologically valid understanding of how children process sounds in real-world environments such as classrooms, where multiple simultaneous demands on cognition and perception occur.

**Figure 1:**
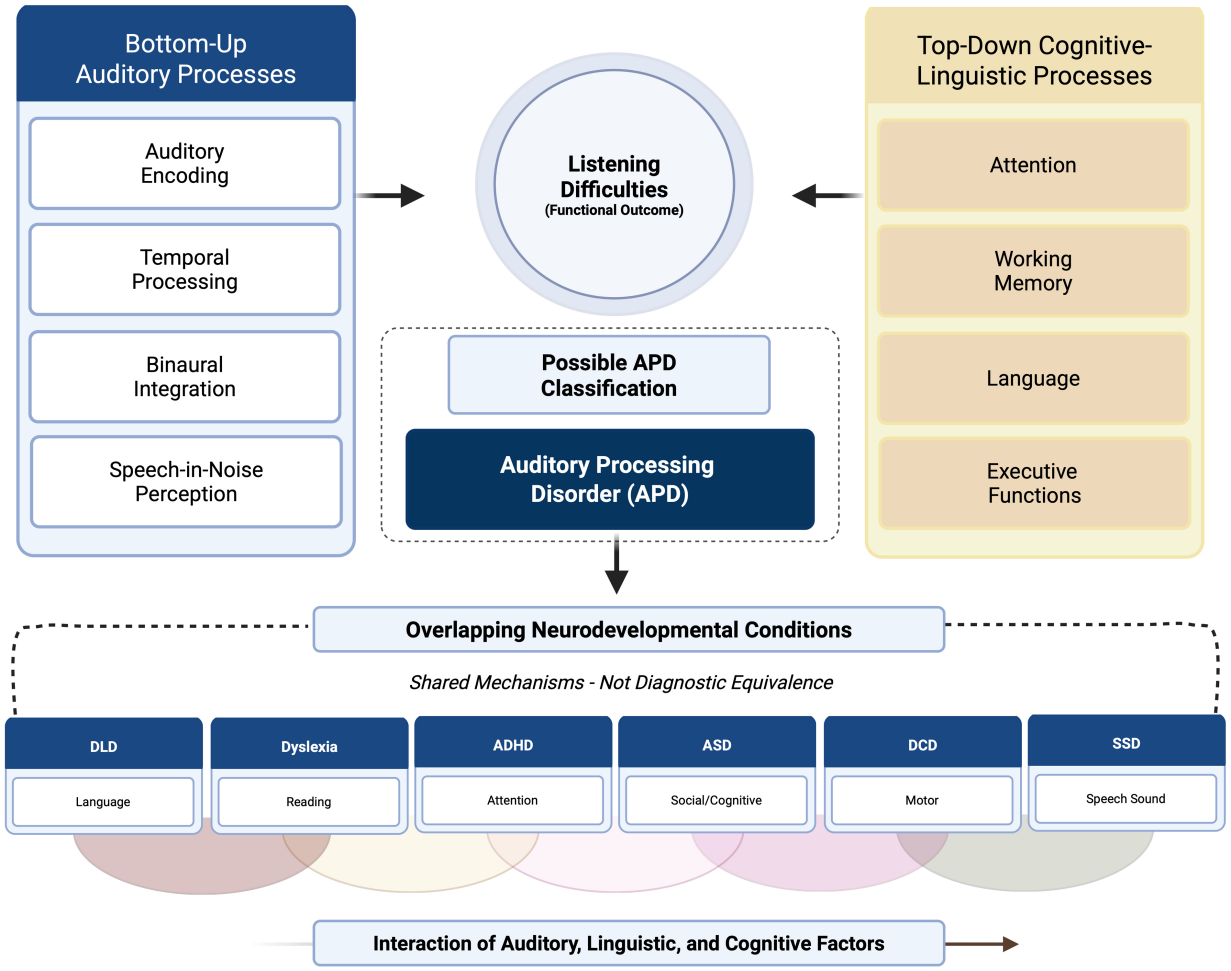
Multidimensional model of listening difficulties in childhood. Bottom-up auditory processes (auditory encoding, temporal processing, binaural integration, and speech-in-noise perception) and top-down cognitive-linguistic processes (attention, working memory, language, and executive functions) converge to influence listening difficulties as a functional outcome. Within this multidimensional framework, auditory processing disorder (APD) represents a possible diagnostic classification rather than a unitary causal entity. The lower panel illustrates overlapping neurodevelopmental conditions that may share underlying mechanisms with listening difficulties without implying diagnostic equivalence. These include developmental language disorder (DLD), dyslexia, attention-deficit/hyperactivity disorder (ADHD), autism spectrum disorder (ASD), developmental coordination disorder (DCD), and speech sound disorder (SSD). The model emphasizes the interaction of auditory, linguistic, and cognitive factors in shaping clinical presentation and highlights the distinction between shared mechanisms and categorical diagnoses.

Clinically, acknowledging the overlap between APD and other neurodevelopmental conditions has important implications. Rather than attempting to isolate a single “auditory” cause, assessment should adopt a multidimensional approach that examines the contribution of attention, language, and memory alongside auditory measures (Neijenhuis et al., 2019). Multidisciplinary evaluation—combining audiologists, speech-language pathologists, psychologists, and educators—enhances diagnostic accuracy and ensures that intervention targets functional communication rather than test-specific deficits. This approach aligns with the growing movement toward functional and interdisciplinary assessment recommended by recent consensus statements [ESLA (European Society for Language and Communication Disorders), 2021; Moore et al., 2025] (see Figure 1).

## 6 Lack of international consensus and guideline limitations

Despite more than four decades of research and multiple position statements, no international consensus has yet been achieved regarding the definition, diagnosis, or management of Auditory Processing Disorder (APD) in children (Jerger, 2009). The persistence of conceptual fragmentation across professional bodies has resulted in significant clinical heterogeneity and confusion among practitioners, educators, and families (DeBonis, 2015; Neijenhuis et al., 2019; Dawes and Bishop, 2009). Differences among national guidelines—such as those of the American Speech-Language-Hearing Association (ASHA) (2005), American Academy of Audiology (2010), and British Society of Audiology (2018)—extend beyond test recommendations to encompass divergent views about the very nature of APD as a diagnostic entity (Schow et al., 2020).

A major limitation across these documents is the lack of methodological transparency in how recommendations were derived. Few guidelines explicitly describe their evidence appraisal process, and most rely primarily on expert consensus rather than systematic review. The AGREE II instrument (Brouwers et al., 2010) and the Institute of Medicine (IOM) Standards for Developing Trustworthy Clinical Practice Guidelines (2011) identify six key dimensions of quality: (1) scope and purpose, (2) stakeholder involvement, (3) rigor of development, (4) clarity of presentation, (5) applicability, and (6) editorial independence. When evaluated against these standards, most APD-related guidelines show deficiencies in at least four domains—particularly in methodological rigor, conflict-of-interest disclosure, and applicability to diverse linguistic or cultural settings.

Some recent efforts, such as the ESLA (European Society for Language and Communication Disorders) (2021) (Drosos et al., 2024) consensus, have begun to integrate linguistic, cognitive, and contextual factors into APD assessment. However, even these updated frameworks still lack formal evidence grading and external peer review. In contrast to other clinical fields where guideline development follows systematic evidence synthesis (e.g., audiology standards for hearing aids or tinnitus), APD guidance remains largely descriptive and experience-based. This gap undermines reproducibility, creates inconsistencies in service delivery, and limits cross-national comparability of prevalence data.

The absence of international harmonization has direct implications for diagnosis and treatment. In some countries, the use of rigid diagnostic cut-offs (e.g., ≥2 SD below the mean on two tests) leads to potential overdiagnosis and inappropriate labeling. In others, the lack of standardized protocols contributes to underdiagnosis, with children experiencing listening difficulties but not meeting formal criteria for APD. Moreover, the scarcity of culturally adapted normative data exacerbates inequities in access to services, especially in multilingual or low-resource contexts (Neijenhuis et al., 2019; Moore et al., 2025).

To move forward, future consensus initiatives should adopt a transparent, evidence-based, and participatory approach. This would include:

(1) Systematic literature review to inform recommendations;
(2) Formal use of quality appraisal tools such as AGREE II;
(3) Stakeholder engagement involving clinicians, educators, and families; and
(4) Alignment with ethical and cross-cultural considerations. Establishing internationally recognized diagnostic benchmarks would not only enhance scientific credibility but also support equitable clinical practice and policy-making. As emphasized by (Moore et al., 2025; Dawes and Bishop, 2009), a unified framework should prioritize functional outcomes and ecological validity over rigid test-based classifications.

## 7 Clinical, educational, and ethical implications

The lack of diagnostic consensus and the psychometric limitations of existing tests have profound implications for clinical practice. Children referred for Auditory Processing Disorder (APD) assessment often present heterogeneous profiles that extend beyond auditory processing, including co-occurring difficulties in language, attention, and learning (Sharma and Purdy, 2020; Moore et al., 2025). This heterogeneity challenges clinicians to differentiate between genuine auditory dysfunction and broader neurodevelopmental variability.

In many clinical contexts, reliance on rigid diagnostic thresholds, such as “≥2 SD below the mean on two tests”, has led to a risk of overdiagnosis. Such categorical classification can divert resources toward labeling rather than functional support, with limited evidence that diagnostic assignment itself improves outcomes (DeBonis, 2017a,b). Conversely, underdiagnosis may occur when children with listening difficulties fail to meet formal APD criteria but nonetheless experience real communicative or academic challenges. Both extremes highlight the need for individualized, evidence-based evaluation that integrates audiological, cognitive, and environmental perspectives.

In educational settings, the consequences of diagnostic inconsistency are equally significant. Teachers and school-based clinicians frequently face confusion regarding how to interpret APD diagnoses and how best to accommodate affected students (Fey et al., 2011; Sharma et al., 2022). Because APD often overlaps with language and attention difficulties, interventions that target only auditory discrimination are rarely sufficient. Evidence suggests that functional classroom accommodations, such as preferential seating, reduced background noise, clear teacher articulation, and multimodal presentation of information, may improve listening performance and classroom participation when implemented as part of a broader multidisciplinary support plan (Fey et al., 2011; Sharma and Purdy, 2020).

Collaborative frameworks between audiologists, speech-language pathologists, psychologists, and educators are critical. Interdisciplinary planning ensures that intervention goals are functional, focusing on listening comprehension, task engagement, and classroom participation rather than narrowly defined test outcomes. Moreover, parental involvement and teacher feedback provide ecological data that better reflect daily listening demands, bridging the gap between clinical assessment and real-world functioning (Neijenhuis et al., 2019).

The ethical dimension of APD diagnosis centers on the responsible use of diagnostic labels and equitable access to services. Overpathologizing typical developmental variability risks medicalizing normal listening diversity, whereas failing to identify children with genuine auditory processing difficulties denies them appropriate support (Spaulding et al., 2006; Vermiglio, 2014). Clinicians have an ethical obligation to ensure that assessment procedures are transparent, evidence-based, and free from commercial or institutional bias (Brouwers et al., 2010). Furthermore, the absence of culturally adapted norms disproportionately affects children in multilingual or low-resource settings, reinforcing social inequities (Moore et al., 2025). Ethical practice in APD assessment therefore extends beyond individual clinical responsibility to include advocacy for inclusive and culturally sensitive diagnostic frameworks. In line with the IOM (2011) standards, clinicians and researchers must promote transparency in guideline development, conflict-of-interest disclosure, and data sharing to foster public trust and global comparability.

Mitigating these challenges requires a multidimensional approach grounded in interdisciplinary collaboration and ecological validity. Clinical assessment should integrate audiological, linguistic, cognitive, and behavioral data rather than relying exclusively on auditory tests. Functional outcome measures, such as listening questionnaires and teacher or caregiver reports, provide essential information about real-world listening performance and should be prioritized alongside laboratory assessments. Evaluation should also consider the communicative and academic environments in which children operate, emphasizing naturalistic or simulated contexts that capture authentic auditory demands. Before assigning permanent diagnostic labels, trial-based interventions with systematic monitoring of progress can help determine whether difficulties persist despite adequate environmental and educational support. Periodic re-evaluation is equally important, as auditory and cognitive abilities evolve throughout development and schooling. Finally, clinician training and interprofessional communication are essential to ensure consistent interpretation of assessment outcomes across settings. These measures collectively promote diagnostic integrity, reduce misclassification, and align APD assessment with ethical, educational, and developmental principles.

## 8 Discussion and future directions for functional and interdisciplinary assessment

Auditory Processing Disorder (APD) in childhood remains a construct characterized by conceptual ambiguity, diagnostic variability, and methodological inconsistency. Across the preceding sections, this review has shown that the field continues to rely on behavioral test batteries with limited psychometric robustness, lacking both cross-cultural normative data and ecological validity. The absence of international consensus, combined with the overlap between APD and other neurodevelopmental conditions, has contributed to diagnostic fragmentation and inconsistent clinical practice worldwide (Neijenhuis et al., 2019; Samara et al., 2023; Moore et al., 2025).

Collectively, these challenges point to the need for a paradigm shift—from a test-centered approach to a functional, interdisciplinary model of listening assessment. Rather than viewing APD as a discrete auditory disorder, contemporary evidence supports its conceptualization as a multidimensional listening difficulty, arising from the interaction of auditory, linguistic, attentional, and cognitive processes (Fey et al., 2011; Sharma and Purdy, 2020). A functional approach emphasizes the child’s real-world listening behavior and participation, integrating clinical measures with ecological and developmental perspectives.

### 8.1 Toward an integrative assessment model

Based on current evidence, a stepwise and interdisciplinary framework for evaluating listening difficulties in children can be outlined as follows. The process begins with screening for peripheral hearing integrity, using pure-tone audiometry, tympanometry, and otoacoustic emissions to exclude peripheral loss. When hearing thresholds are normal, clinicians should proceed to a comprehensive case history and listening profile, incorporating caregiver and teacher questionnaires to capture contextual information about everyday listening behaviors.

Next, an interdisciplinary triage should be conducted, where audiologists, speech-language pathologists, and psychologists collaboratively determine the likely contribution of auditory, language, or attentional factors. If attention or language difficulties are primary, these should be addressed first, followed by reevaluation of auditory function. When an auditory component is suspected, targeted auditory measures, such as dichotic listening, temporal processing, or speech-in-noise tasks, may be administered, interpreted cautiously in light of developmental and cognitive context (Tomlin et al., 2015; Sharma et al., 2022).

Intervention planning should be individualized, combining environmental accommodations, cognitive-linguistic support, and auditory training where appropriate. Importantly, all interventions should include functional outcome monitoring to assess real-world improvement, using validated measures such as classroom listening questionnaires, academic performance indicators, and parental feedback. Periodic reassessment should be standard practice, acknowledging that listening and cognitive abilities evolve across development and educational experiences (Moore et al., 2025).

### 8.2 Evidence-based interventions and translational opportunities

Despite the controversies surrounding APD diagnosis, several interventions show promise when applied within a functional, individualized framework. Systematic reviews indicate modest but meaningful gains in listening and language outcomes from auditory training programs, particularly when paired with linguistic or attentional components (Fey et al., 2011). Classroom-based environmental modifications, including preferential seating, assistive listening devices, and optimized acoustic conditions, are widely recommended to support listening comprehension and classroom participation, particularly when integrated with broader educational and communication strategies (Fey et al., 2011; Sharma and Purdy, 2020). Moreover, metacognitive strategies that teach children to actively monitor and manage their listening behaviors can promote generalization beyond the therapy setting (Sharma et al., 2022). These multimodal interventions, though heterogeneous, underscore the importance of ecological and interdisciplinary design over isolated auditory stimulation paradigms.

### 8.3 Limitations and future directions

This review has several limitations inherent to its narrative design. Although care was taken to conduct a comprehensive and structured literature search, the absence of formal meta-analytic synthesis introduces the possibility of selection bias. The heterogeneity of APD definitions and diagnostic practices across studies further limits the comparability of results. Moreover, most available evidence originates from high-income, monolingual populations, reducing generalizability to diverse linguistic and cultural contexts. Future research should address these gaps through large-scale, longitudinal, and cross-linguistic studies that examine how auditory, cognitive, and environmental factors interact over time.

Moving forward, the field must prioritize transparent, evidence-based, and inclusive guideline development. Collaborative networks that integrate audiology, cognitive neuroscience, education, and developmental psychology will be essential to establish unified diagnostic benchmarks and intervention pathways. As research converges on the interplay between auditory perception and higher-order cognition, a more coherent, interdisciplinary understanding of listening difficulties may emerge—one that transcends categorical boundaries and translates scientific insights into equitable, real-world benefits for children and families.
