# Correction: Auditory Processing Disorder in childhood: a critical appraisal of diagnostic validity, functional assessment, and interdisciplinary practice

**DOI:** 10.3389/fnhum.2026.1930059

**Published:** 2026-08-03

**Authors:** Júlio César Claudino dos Santos, Marco Antonio Arruda, Marcelo Rodrigues Masruha

**Affiliations:** 1 Laboratório de Neurologia Experimental, Programa de Pós-graduação em Ciências da Saúde, Universidade do Extremo Sul Catarinense, UNESC, Criciúma, Brazil; 2 Universidade Federal Fluminense (UFF), Niterói, Rio de Janeiro, Brazil; 3 Programa de Pós-Graduação em Neurologia e Neurociência, Universidade Federal Fluminense, UFF, Niterói, Brazil; 4 Instituto Glia – Neurologia e Neurodesenvolvimento, Ribeirão Preto, Brazil; 5 Instituto de Neurociência do Espírito Santo, Vitória, Brazil

**Keywords:** auditory perceptual disorders, child, diagnosis, evidence-based practice, neurodevelopmental disorders

The reference for Moore (2024) was erroneously written as:

Moore, D. R. (2024). *Relation between research findings and clinical practice in* *childhood listening difficulty and auditory processing disorder*. J. Speech Lang. Hear. Res. 67, 2978–2991. doi: 10.1044/2024_JSLHR-23-0074.

It should be:

Moore, D. R., Lin, L., Bhalerao, R., Caldwell-Kurtzman, J., and Hunter, L. L. (2025). Multidisciplinary clinical assessment and interventions for childhood listening difficulty and auditory processing disorder: relation between research findings and clinical practice. *J.* *Speech Lang. Hear. Res*. 68, 2978–2991. doi: 10.1044/2025_JSLHR-24-00306.

The reference for Tomlin et al. (2015) was erroneously written as:

Tomlin, D., Dillon, H., Sharma, M., and Rance, G. (2015). *The impact of auditory* *processing and cognitive abilities in children*. J. Am. Acad. Audiol. 26, 50–61.

It should be:

Tomlin, D., Dillon, H., Sharma, M., and Rance, G. (2015). The impact of auditory processing and cognitive abilities in children. *Ear Hear*. 36, 527–542. doi: 10.1097/AUD.0000000000000172.

The reference for DeBonis (2017a) was erroneously written as:

DeBonis, D. A. (2017a). *It is time to rethink central auditory processing disorder* *protocols for school-aged children*. Am. J. Audiol. 26, 252–264.

It should be:

DeBonis, D. A. (2015). It is time to rethink central auditory processing disorder protocols for school-aged children. *Am. J. Audiol*. 24, 124–136. doi: 10.1044/2015_AJA-14-0037.

The citation to Bishop et al. (2022) has been replaced by the following reference:

Dawes, P., and Bishop, D. V. M. (2009). Auditory processing disorder in relation to developmental disorders of language, communication and attention: a review and critique. *Int. J. Lang. Commun. Disord*. 44, 440–465. doi: 10.1080/13682820902929073.

The citation to Keith et al. (2023) has been replaced by the following reference:

Dawes, P., and Bishop, D. V. M. (2009). Auditory processing disorder in relation to developmental disorders of language, communication and attention: a review and critique. *Int. J. Lang. Commun. Disord*. 44, 440–465. doi: 10.1080/13682820902929073.

The citation to Iliadou et al. (2023) has been replaced by the following reference:

Samara, M., Thai-Van, H., Ptok, M., Glarou, E., Veuillet, E., Miller, S., et al. (2023). A systematic review and metanalysis of questionnaires used for auditory processing screening and evaluation. *Front. Neurol*. 14:1243170. doi: 10.3389/fneur.2023.1243170.

The citation to Hämäläinen et al. (2022) has been replaced by the following reference:

Dawes, P., and Bishop, D. V. M. (2009). Auditory processing disorder in relation to developmental disorders of language, communication and attention: a review and critique. *Int. J. Lang. Commun. Disord*. 44, 440–465. doi: 10.1080/13682820902929073.

A correction has been made to Section 5, **“Neurocognitive overlap with other** **developmental disorders.”**

Original text:

“Functional MRI and electrophysiological studies show that children with APD and those with DLD or dyslexia often display reduced activation or atypical connectivity in fronto-temporal networks implicated in speech perception, phonological processing, and attention modulation (Hämäläinen et al., 2022; Keith et al., 2023).”

Corrected text:

“Functional MRI and electrophysiological studies suggest that listening difficulties frequently co-occur with alterations in neural systems supporting language, attention, and auditory processing, although the specificity of these findings to APD remains uncertain (Dawes and Bishop, 2009; Sharma and Purdy, 2020).”

A correction has been made to Section 5, **“Neurocognitive overlap with other** **developmental disorders.”**

Original text:

“Emerging models now conceptualize APD as part of a broader continuum of listening and communication difficulties rather than a categorical disorder (Moore, 2018; Iliadou et al., 2023).”

Corrected text:

“Emerging models increasingly conceptualize listening difficulties as multidimensional phenomena involving auditory, linguistic, attentional, and cognitive factors rather than as isolated auditory deficits (Moore, 2018; Dawes and Bishop, 2009).”

A correction has been made to Section 6, **“Lack of international consensus and** **guideline limitations.”**

Original text:

“The persistence of conceptual fragmentation across professional bodies has resulted in significant clinical heterogeneity and confusion among practitioners, educators, and families (DeBonis, 2017a; Neijenhuis et al., 2019; Iliadou et al., 2023).”

Corrected text:

“The persistence of conceptual fragmentation across professional bodies has resulted in significant clinical heterogeneity and confusion among practitioners, educators, and families (DeBonis, 2015; Neijenhuis et al., 2019; Dawes and Bishop, 2009).”

A correction has been made to Section 6, **“Lack of international consensus and** **guideline limitations.”**

Original text:

“As emphasized by recent authors (Moore, 2024; Iliadou et al., 2023), a unified framework should prioritize functional outcomes and ecological validity over rigid test-based classifications.”

Corrected text:

“As emphasized by Moore et al. (2025) and Dawes and Bishop (2009), a unified framework should prioritize functional outcomes and ecological validity over rigid test-based classifications.”

A correction has been made to Section 7, **“Clinical, educational, and ethical** **implications.”**

Original text:

“Evidence suggests that functional classroom accommodations, such as preferential seating, reduced background noise, clear teacher articulation, and multimodal presentation of information, yield greater benefits than auditory training alone (Keith et al., 2023).”

Corrected text:

”Evidence suggests that functional classroom accommodations, such as preferential seating, reduced background noise, clear teacher articulation, and multimodal presentation of information, may improve listening performance and classroom participation when implemented as part of a broader multidisciplinary support plan (Fey et al., 2011; Sharma and Purdy, 2020).

A correction has been made to Section 8, **“Discussion and future directions for** **functional and interdisciplinary assessment.”**

Original text:

“Rather than viewing APD as a discrete auditory disorder, contemporary evidence supports its conceptualization as a multidimensional listening difficulty, arising from the interaction of auditory, linguistic, attentional, and cognitive processes (Bishop et al., 2022; Sharma and Purdy, 2020).”

Corrected text:

“Rather than viewing APD as a discrete auditory disorder, contemporary evidence supports its conceptualization as a multidimensional listening difficulty, arising from the interaction of auditory, linguistic, attentional, and cognitive processes (Dawes and Bishop, 2009; Sharma and Purdy, 2020).”

A correction has been made to Section 8.2, “*Evidence-based interventions and* *translational opportunities*”

Original text:

“Classroom-based environmental modifications—including preferential seating, assistive listening devices, and optimized acoustic conditions—consistently enhance listening comprehension and learning participation (Keith et al., 2023).”

Corrected text:

“Classroom-based environmental modifications, including preferential seating, assistive listening devices, and optimized acoustic conditions, are widely recommended to support listening comprehension and classroom participation, particularly when integrated with broader educational and communication strategies (Fey et al., 2011; Sharma and Purdy, 2020).”

The original version of this article has been updated.

